# Magnesium for Implants: A Review on the Effect of Alloying Elements on Biocompatibility and Properties

**DOI:** 10.3390/ma15165669

**Published:** 2022-08-18

**Authors:** S. Fida Hassan, M. T. Islam, N. Saheb, M. M. A. Baig

**Affiliations:** 1 Department of Mechanical Engineering, King Fahd University of Petroleum and Minerals, Dhahran 31261, Saudi Arabia; 2 Interdisciplinary Research Center for Advanced Materials, King Fahd University of Petroleum and Minerals, Dhahran 31261, Saudi Arabia

**Keywords:** magnesium, implant, biodegradable, alloying element

## Abstract

An attempt is made to cover the whole of the topic of biodegradable magnesium (Mg) alloys with a focus on the biocompatibility of the individual alloying elements, as well as shed light on the degradation characteristics, microstructure, and mechanical properties of most binary alloys. Some of the various work processes carried out by researchers to achieve the alloys and their surface modifications have been highlighted. Additionally, a brief look into the literature on magnesium composites as also been included towards the end, to provide a more complete picture of the topic. In most cases, the chronological order of events has not been particularly followed, and instead, this work is concentrated on compiling and presenting an update of the work carried out on the topic of biodegradable magnesium alloys from the recent literature available to us.

## 1. Introduction

Biodegradable materials for implants have been in clinical use for some time now [1]. These materials have come into prominence in lieu of non-biodegradable, permanent implants, which have had temporary applications to afford the body a healing period after which the implants are no longer required. The use of permanent implants for temporary applications meant a secondary operation is carried out to remove the implant. Apart from the obvious trauma and the inconvenience suffered by the patients and their families, it also meant added medical costs and medical resources are spent in this ordeal, not to mention the economic value of time spent by all parties involved. These permanent implants were mostly either titanium or steel-based alloys. Common issues of the resulting ‘effects’ by the permanent orthopedic implants include inflammation, infection, stress shielding, and consequent bone loss. Stress shielding is due to the higher stiffness of the implants, leading to the dis-use of adjacent bones and further leading to a gradual loss of bone structure and weakening of the bones. The solution has been the use of biodegradable materials for implants, which would corrode naturally within the body, after or during affording the required time for the healing. Biodegradable materials such as biodegradable polymers (with polyglycolic acid (PGA) and polylactic acid (PLA) being most common), bioceramics (Tricalcium phosphate (TCP), Hydroxyapatite (HA)), and biodegradable Mg alloys [2] have been in increasing use for this reason. The applications included bone fixtures such as nails [3], screws [4], clips [5], wires [6], and stents [7]. Of interest has been applications involving bones, which is where magnesium and magnesium-based alloys have been researched dominantly.

Magnesium [8] has the electron configuration of 1s^2^2s^2^2p^6^3s^2^ [8], with a hexagonal close-packed (HCP) crystal structure. At 1.7 g/cm^3^ it has the lowest density amongst all structural metals. It has a Young’s Modulus of approximately 45 GPa. In comparison, cortical bone has a density of 1.8–2.0 g/cm^3^ and a Young’s modulus of 7–30 GPa [9]. This closeness in properties is an advantage for which magnesium and its alloys have been of significant research as a biodegradable implant material.

Magnesium has been found to have special osteoconductive properties, which is much appreciated when used as bone implants. It has been reported that Mg, as a cofactor of the alkaline phosphatase isozymes, helps in the healing and remodeling of the bone tissue [10]. Magnesium-based stents are also useful where biodegradable nasal stents could help avoid treatment failure that occurs due to the secondary operations that are required of traditional implants [11]. In the dental field, the use of scaffolds made of Mg/PLGA porous composites to improve bone healing following tooth extraction has been used [12]. More recently, in the dental and orthopedic fields, Mg in bone cement composites have been used to obtain high-strength cement with both biodegradability and bioactivity in the form of magnesium calcium phosphate/sodium alginate composite cement [13].

Others still have used Mg in magnesium/calcium phosphate cements to induce an improved cellular response of bone marrow stromal cells (BMSCs) [14]. Mg ions have been known to promote osteogenic activity of bone marrow stromal cells [15]. Furthermore, various studies have been conducted to observe the effect of Mg alloys on mesenchymal stem cells [16,17]. B. Kanter et al. [18] has demonstrated the suitability of magnesium phosphate cements in partially load-bearing defects of a sheep model. However, Magnesium phosphate is also one of the mineral components of kidney stones [19], although any potential relationship between that and the biodegradable Mg-based cement is not clear.

The potential application of biodegradable magnesium alloys is not limited to its use as temporary implants. Research has been carried out to investigate the utilization of the fast degradation characteristics of magnesium with aqueous media, or in this case, blood, to provide the thrust in magnesium-based micromotors [20]. Moreover, research into a magnesium-based biodegradable battery has been conducted [21] to investigate the potential applications to power bioresorbable transient implants.

There has also been a number of research studies carried out to improve the surface properties of the traditional implants with the use of magnesium to achieve better performance. These include the co-implantation of Zn/Mg ions on titanium dental implant surfaces to improve osteogenesis [22] properties of the implant, and the Mg ion implantation on micro and nano-structured titanium surfaces to improve its osteogenic differentiation and proliferation of bone marrow cells [23] so as to achieve greater contact of the corroding implant and the healing bone tissue.

With all this being said, the effect of the individual alloying elements on the biocompatibility, biodegradability, microstructure, and mechanical properties of pure magnesium need to be understood clearly and is therefore discussed in detail in this study, and the effects of work history, heat treatment, and coating have been briefly discussed.

## 2. Requirements for Bio Application of Mg Alloys and Their Corrosion Strengths

The biocompatibility of magnesium biodegradable alloy is determined via cytotoxicity tests conducted either in vitro or in vivo. The cytotoxicity tests are mainly designed to test for either the cell proliferation in a given medium of magnesium-degraded products or cell adhesion to the magnesium alloy being tested. The standard of the in vitro tests is usually performed as in standard ISO 10993 part 5 [24,25,26,27].

However, there has been poor correlation between in vitro and in vivo studies when using the ISO 10993 standard [24,27] due to which, in recent years, there have been studies to propose a modified approach to the standards of in vitro testing of cytotoxicity. Some researchers suggested dilution of the extracts to achieve more accurate in vitro tests. J. Wang et al. [24] proposed a 6- to 10-times dilution of extracts while J. Fischer [25] proposed a 10-times dilution for as-cast material. Others, such as L. Scheideler [27], proposed the use of a bovine serum instead of the standard recommended cell culture medium. However, X. Liu et al. [28] studied the extraction parameters for its influence in the predictability of in vivo tests via in vitro tests and they specifically discounted the addition of bovine serum albumin (BSA) and fetal bovine serum (FBS) for greater predictability because of their acceleration of corrosion of magnesium samples during extraction and their effects on cell health during the test. Their proposed solution was the establishment of a database recording the tolerance of cells towards the main hazards of metal ions, pH, and H_2_ gas along with a set of in vitro corrosion tests with high similarity to in vivo tests.

O. Jung et al. [29] reported an optimized procedure for testing the in vitro cytocompatibility, within the standard DIN EN ISO 10993-5:2009. Their study recommended the use a combination of an indirect assay and a complementary direct live-dead staining of cells grown on magnesium materials, for testing cell proliferation, viability, and cytotoxicity. It was also suggested that the exposed surface area of Mg in the in vitro testing media may have played a role in the discrepancies in results. This is also a possible reason for the differences between in vitro and in vivo cytotoxicity results discussed above. A higher exposure of the magnesium surface has been recommended to give better results [29].

The release of gases to the surrounding vicinity, i.e., tissue [30] and bone, is investigated to determine its influence and effects. Slower degradation rates are necessary for low gas evolution rates. Higher rates of evolution of H_2_ gas could lead to inflammation and swelling of the surrounding tissue. Similarly, gas pockets and the pressure associated with the release of H_2_ could lead to deformations during the osteogenesis applications of magnesium alloys.

Due to the above-mentioned limitations, only those elements with positive or negligibly negative effects to the human body should be considered for use as in applications involving biodegradable magnesium alloys. The requirements for bio application of the magnesium alloys are (i) uniform corrosion degradation, (ii) a slow and controlled degradation rate, and (iii) good cytotoxicity

## 3. Biotoxicity of Common Alloying Elements

### 3.1. Toxicology and Pathophysiology

The nutritional requirements of the human body and therefore, by extension, the tolerance of the body to different elements, vary by ethnicity—which can be said to be differentiated by geography and culture. For instance, it has been reported by the World Health Organization (WHO) [31] that there exists wide differences in dietary intake of calcium levels between the different regions of the world, which has been attributed to the different diets based on food culture and availability. This is an indication of the differences in allowances when considering each specific group of people. Therefore, this variation in tolerance needs to be respected when choosing the magnesium alloy for use as implants. The degradation rate should be within these tolerances to avoid unnecessary complications.

The nature of the chemical reaction and thereby the toxicity of the alloying elements is dependent on identifying the speciation of the elements in the extracellular fluid, which is the medium of their transport to various other locations. This is, however, a difficult task as the metals in the biological environment could form complexes with any of the available potential ligands and as many of the metal complexes are kinetically labile, i.e., susceptible to being altered [32]. For this reason, the toxicology of the alloying elements will be discussed on the basis of their general toxicology and nutritional value rather than their specific effects on individual species. Moreover, for the purpose of relevancy to the topic, the toxicology of the inhalation route of exposure to the metals is not discussed.

#### 3.1.1. Magnesium (Mg)

The human skeleton contains approximately 50–60% of the body’s magnesium, with 1% in the extracellular fluid and the remaining in the muscles and tissues [31]. It is also an essential element involved in the regulation of potassium fluxes and the metabolism of calcium [31]. The magnesium forms a surface constituent of the hydroxyapatite mineral component in the bone and acts in the regulation of magnesium content in the serum at times of deficiency. At times of plenty in erythrocyte magnesium, the bone mineral density increases, while in times of deficiency, it helps make up the magnesium amount to a certain extent, and this form of accessible magnesium availability has been reported to decrease significantly with age [31].

#### 3.1.2. Iron (Fe)

Iron is an essential element for the human body [33], and it is one of the most abundant metals in the body. Iron is essential for oxygen transport (Hemoglobin) and cellular functions such as the synthesis of RNA and DNA, as well as the synthesis of proteins, and is also involved in the regulation of gene expression among many other functions [33]. Additionally, low levels of Iron in the body have also been known to cause anemia. However, an excess of iron, or an iron overload, has also been reported to lead to abnormal interference in the body and cause serious health issues including iron complications arising from altered iron content in cells and tissues, leading to death if left untreated [33].

#### 3.1.3. Calcium (Ca)

Calcium helps provide rigidity to the human skeleton, and its ions are involved in many aspects of the body’s metabolism [31]. It is the fifth most abundant element in the human body and constitutes approximately 2% of an adult’s lean body mass [31], of which almost all are found in the human skeletal system including the teeth and the soft tissues. Approximately 0.1% of the total Calcium in the body is available in the extracellular fluids and are present in the form of ions (1.20 mmol/L or 4.8 mg/100 mL) and complexes (1.6 mg/100 mL or 0.4 mmol/L [31]. Low levels of Ca are regulated via bone resorption, and a higher intake of Ca is absorbed back while the excess unabsorbed Ca is excreted out in the feces [31]. The bone mineral serves as a reservoir in this process. However, very high doses of Ca in carbonate form have been reported to lead to the precipitation of Ca salts in the renal tissue, while Ca deficiency leads to osteoporosis [31].

#### 3.1.4. Zinc (Zn)

Zinc is an essential element, with its presence in all body tissues and fluids, though the plasma zinc accounts for only approximately 0.1% of the total zinc content in the body. It is essential for the enzymes involved in the metabolism of nutrients such as carbohydrates, lipids, proteins, as well as nucleic acids [31]. Furthermore, it is even essential for genetic expression and helps to stabilize the molecular structures of membranes and other components of the cells. Zn has a concentration presence of 0.46 μmol/g (30 μg/g) in the lean body mass [31]. The plasma zinc is regulated via homeostatic control.

#### 3.1.5. Copper (Cu)

Cu is essential and is the third most abundant trace element in the human body [34,35]. Cu^2+^ affects gene expression in mammals [36]. It is an important catalyst for the synthesis of heme and absorption of iron [34]. The body commonly obtains it via ingestion, i.e., as water contaminants or as nutritional components of food. This pathway of absorption of Cu into the body is well-regulated, and most of the follow-up reactions are well-documented [35]. However, when used in biodegradable material, the cellular tissues could be exposed to free Cu ions, which, at concentrated levels, could lead to cellular damage due to its inherent highly reactive nature. Although transmembrane transporters and metallochaperones exist to control the levels of intra cellular copper [35], care should be taken in designing implants so as not to dissociate too much Cu into the surrounding contact fluid. Freely available Cu can be potentially toxic if it oxidizes lipids and proteins leading to the formation of intracellular and extracellular toxic free radicals [36]. Copper toxicity is rare, and when it does occur, it primarily affects the liver [34]. Above 3 mg/L of the whole blood concentration of Cu it is reported to lead to gastrointestinal symptoms of toxicity [34].

#### 3.1.6. Silicon (Si)

**Figure 1:**
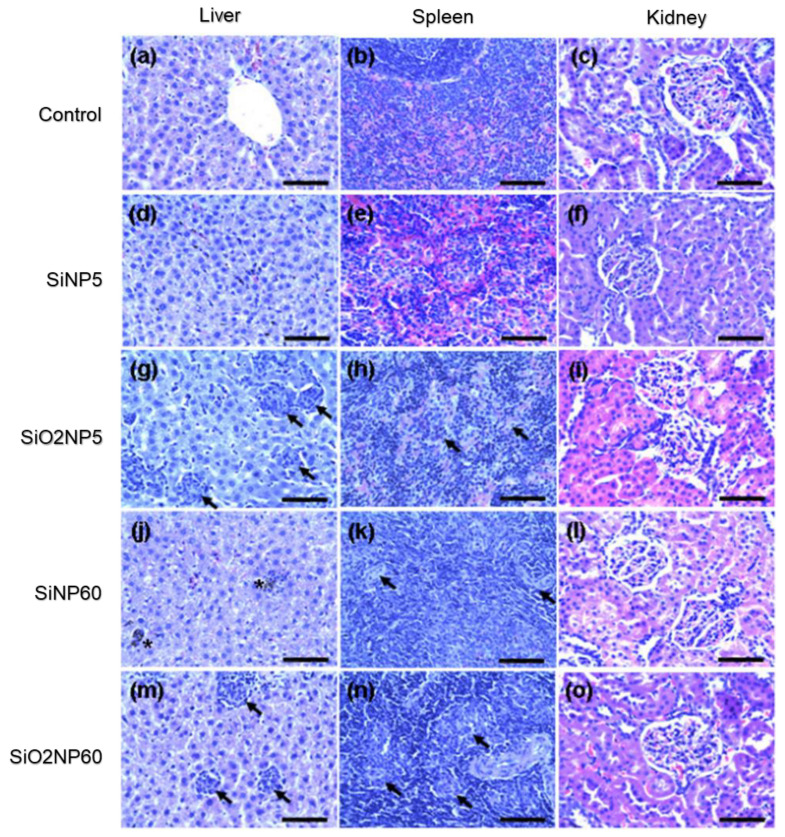
Histological evaluation of organs from rats treated with SiNPs or SiO2NPs. Liver, spleen, and kidney samples were collected at 5 and 60 days after intravenous administration of SiNPs or SiO2NPs at a dose of 7 mg/kg and fixed with paraformaldehyde, followed by staining with hematoxylin and eosin. (**a**–**c**) Vehicle-treated animals (controls); (**d**–**f**) 5 days after SiNP treatment (SiNP5); (**g**–**i**) 5 days after SiO2NP treatment (SiO2NP5); (**j**–**l**) 60 days after SiNP treatment (SiNP60); and (**m**–**o**) 60 days after SiO2NP treatment (SiO2NP60). The arrows indicate granulomas in the liver and spleen. The asterisks indicate microgranulation in the liver. The tissue sections were observed under a microscope at 400×. The scale bar is 25 μm for all images. The pictures are representative of at least four independent sections [42].

Forrest H. Nielson [37] has reported circumstantial evidence that Silicon is an essential nutrient for the human body. Its deficiency has been reported to cause abnormal metabolism of connective tissue and bone in animal tests. Furthermore, tests conducted on rats have shown that Si helps to avoid the accumulation of Al in the brain—which has been connected to Alzheimer’s [37]. Moreover, Si as a polymer, in the form of polydimethysiloxane, has been a popular breast implant material for some time now. However, though these Silicones have been thought to be biologically inert, they have caused inflammation and other complications over time [38,39,40,41]. On the other hand, the intravenous administration of Si nanoparticles has reportedly resulted in relative biocompatibility as far as acute toxicity was concerned but showed granuloma formation (See Figure 1), indicating inflammation in reticulo-endothelial organs such as the liver and spleen [42]. Therefore, the use of elemental Si in implants needs more research, and if it is included as an alloying element to Mg, it must be included cautiously.

#### 3.1.7. Tin (Sn)

Sn is not regarded as an essential element, although multivitamin and mineral supplements reportedly contain up to 0.01 mg in a daily recommended dose [43]. Sn is mostly ingested into the body via canned food consumption, where the cans are lined with or is made of Sn. At a high dosage, Sn accumulates in the bones, liver, and kidney, with bones being the primary site of deposition when Sn is injected intramuscularly and during extended exposure to Sn even through other means such as ingestion [43].

#### 3.1.8. Manganese (Mn)

Mn is an essential element for the human body, which is regulated via homeostasis where excess Mn is mainly excreted via bile and feces. The portion that forms conjugates with the bile is also ultimately excreted out mostly via feces and only a small amount is excreted through urine. It is mainly concentrated in the liver, pancreas, and kidney, but notably, it has its lowest concentrations in fat and bones, wit the latter being of significance in the aspect of its effect in the current discussion. A Mn overload could affect the motor and cognitive abilities of the central nervous system, while prolonged Mn deficiencies have been reported to lead to greater occurrence of symptoms related to Parkinsonism [44].

#### 3.1.9. Aluminum (Al)

Aluminum is rated as Generally Regarded As Safe (GRAS) by the US FDA [32], due to which it is widely available in many foods and medications. Previous studies have reported the lack of proof for Al accumulation in the brain [32]. However, it has been associated with neurotoxicity in recent years and has been reported to lead to Alzheimer’s disease, although there is still skepticism with this conclusion [45].

#### 3.1.10. Lithium (Li)

Lithium is a non-essential trace element. Due to it is similarity with Sodium and Potassium, it can cross all biological barriers, and since it is not protein-bound, it is excreted by the kidneys and does not accumulate in the tissues too much. However, long-term use of Li in therapeutic medicine has been recorded to show toxic effects on the kidney, thyroid, and the Central Nervous System [46]. Taking into account the biodegradable nature of the implants, and the excretion of the Li from the body, the long-term exposure effects can be disregarded, and as such, Li is potentially useable as an alloying element of Mg pending further site-specific tests.

#### 3.1.11. Nickel (Ni)

There is reportedly circumstantial evidence of Ni as an essential element [37], where it is necessary for certain activities of metabolism, and in animal studies conducted, its deficiency has been shown to show negative effects on growth, reproductive performance, and plasma glucose [37]. Ni deficiency has been also reported to affect the distribution of other essential elements in the body such as Ca, Zn, and Fe. However, exposure of Ni in some of the forms other than oral administration have been reported to be a potential source of cancer in animals and humans [37]. It has been suspected of interfering in the functions of Vitamin B12 and Folic acid [37].

#### 3.1.12. Indium (In)

Indium is a non-essential element that produces wide-ranging toxic effects depending on its form. Ionic Indium led to renal failure upon its concentration in the kidney. It is mainly excreted in the urine. The colloidal form of Indium causes damage to the liver and spleen, and this form is mostly excreted via feces [47]. If poorly absorbed when ingested, indium is mostly stored in muscles, skin, and bones [47].

The toxicology of some of the common Mg alloying elements along with their daily allowances as well as the whole blood levels (based on French, Swedish, and Benin populations) and the blood serum levels (based on French and Swedish populations) are given in Table 1 below. The data are as that reported for adults, and it may vary for different age groups. Notably, the reported Mg daily allowance limits from two sources are very different from each other. Again, this could be due to differences in the population, which was considered in the literature. 

### 3.2. In Vitro Biotoxicity

In vitro testing has been carried out in laboratory facilities with the Mg alloy specimens immersed in a cell culture of preferred contents and tested for the cell viability and cytotoxicity after the test duration. Different groups have used a number of different cell mediums in this regard. Biotoxicity is measured in terms of cell viability (%), hemolysis, % and cell adhesion. These parameters are dependent on culture time. It is perhaps noteworthy to mention that the characteristic rates of these parameters need careful investigation when designing the implant itself. This is due to the non-linearity of these parameters. As per the ISO 10993-5:2009, cell viability below 30% is deemed as cytotoxic in nature [60].

**Cytotoxicity:** Measured by Relative Growth Rates (RGR) of the cells as determined by a method such as gradation optical density measurements [61]. The graded scale is used as a means of determining the cytotoxicity of the alloy to the tested cell line.

**Cell viability:** Viability of the cell can be measured by determining the count of live and dead cells. This has been performed by exposing the experimental cell line to the aqueous extract of the alloy [61].

**Cell Adhesion:** This is useful to measure the BIC or the Bone-to-Implant Contact of the Mg alloy, useful in osteogenesis applications. A greater BIC score is useful to avoid the formation of voids between the degrading implant surface and the forming bone tissue.

When the Mg alloys are tested in vitro for cell viability, a range of cell cultures are available for use. The type of cells used depend mostly on the type of design application of the alloy. For instance, those relating to osteosynthesis would be tested with hBMMSCs (human bone marrow mesenchymal stem cells) [4] or MC3T3-E1 (osteoblasts) [62], or U-2OS (human osteosarcoma cells) [63] have been used when testing for osteosynthesis potentiality. Similarly, those that may be exposed to blood or lymph vessels, as is the case of the dissolute ions in the blood, could be tested using VSMC (rodent vascular smooth muscle cells) [62] for animal models. Table 2 has been composed using such in vitro cytotoxicity results of the alloy elements themselves or as binary alloys of Mg in extract media as found in various literature. The elements’ effect on various cell lines are listed along with a summary of the test result. It has to be noted that the cytotoxicity may not always be detected during the period of either in vitro or in vivo tests and could be due to the absence of degradation products’ effects on the specific organs tested in vivo/ex vivo, and this by no means can be taken as having no adverse effect of any alloying elements on the body. Rather, in the case of alloys containing specifically toxic elements in terms of both the toxicity and pathophysiology, it is the view of the Authors that a better way is to refer to the biological aspects of element absorption, accumulation, and excretion pathways during in vivo studies (see Table 3). 

### 3.3. In Vivo Biotoxicity

#### 3.3.1. Clinical Trials: Animal Tests

**Figure 2:**
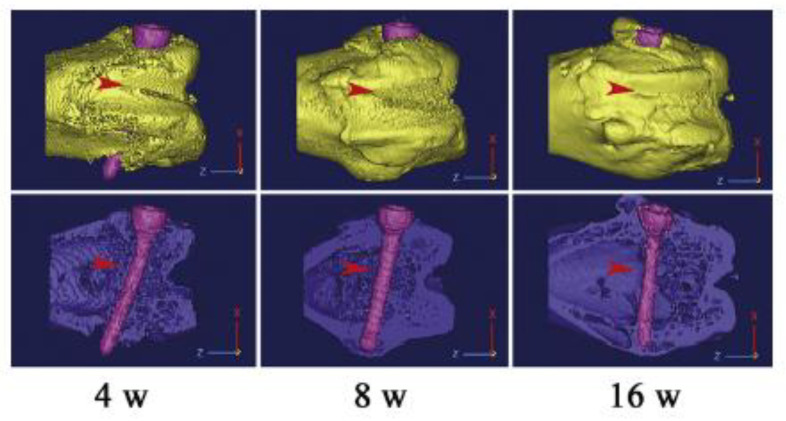
3D images of the gross morphology (**upper**) and coronal sections (**lower**) of femoral intracondyle fixed by HP Mg screws at 4, 8, and 16 weeks. Red arrowheads mark the specific screw portion exposed to fracture gap [72].

Recently, J. Zhang et al. [70] studied the follow-up of an Mg alloy stent [71] in a Rabbit model and reported the ion diffusion and replacement that takes place after the Mg alloy stent was completely degraded, at 20 months after implantation. In vivo tests conducted on a rabbit model by P. Han et al. [72] using high-purity magnesium screws determined some qualitative aspects of the mechanical properties of the screws. As shown in Figure 2, the screw used as a fixature for the femoral fracture with a gap of 3 mm was seen to bend at 4 weeks post-operation. This localized mechanical stress has been observed to lead to a localized corrosion which was detected at 16 weeks. However, these effects were concluded not to have an observable difference in the osseointegration and the overall corrosion behavior of the implant, and during this period, bone tissue formation and fracture healing were also observed, leading to increased prospects of HP Mg-based implants for load-bearing bone fractures.

Recently, H. Bai et al. [73] tested Mg3Zn0.2Ca clips both in vitro and in vivo and reported its suitability as a surgical hemostatic clip achieving complete closure of the blood vessel tested. The clip produced by hot-extrusion followed by blanking and subsequent annealing was found to be biocompatible with no adverse effects 2 weeks after degradation, with a uniform degradation.

#### 3.3.2. Clinical Trials: Human Tests

**Figure 3:**
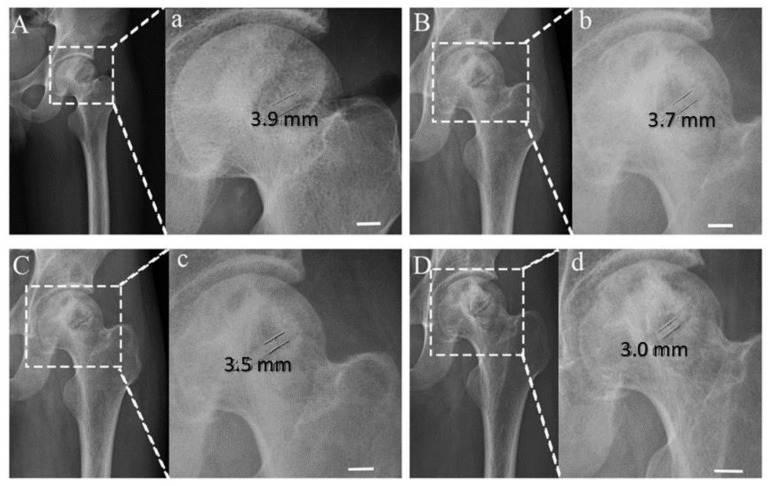
The temporal changes in biodegradation rate of Mg screws interpreted by a decrease in screw diameter. (**A**–**D**) X-ray imaging of femoral head in patients implemented with Mg screws at 1 (**A**), 3 (**B**), 6 (**C**), and 12 (**D**) months postoperatively. (**a**–**d**) Magnified surgical regions in (**A**–**D**) for measurement of screw diameter at different time points. Scale bar represents 10 mm [74].

D. Zhao et al. [74] has conducted a clinical trial on the application of Mg alloy screws in fixing the bone grafts during hip-preserving surgery as shown in Figure 3. The method was used to overcome the slight slip and displacement, which may occur otherwise and has claimed significant improvement in the Harris Hip Score (HHS) used as a measure of the rate of recovery. Moreover, they did not find any adverse reactions on the bone tissue around the screws via CT imaging and the levels of degradation products in the serum postoperative was found to be same as the control and within the allowable limits.

In 2013, an Mg alloy consisting of MgYREZr and industrially trademarked as MAGNEZIX^®^ was clinically tested for use in hallux valgus surgery as screw implants and found to be similar to currently popular titanium screws both radiographically and clinically [75]. The hollow screws (Ø2 mm and Ø1.3 mm cannulation) of MAGNEZIX^®^ were made using the Powder Metallurgy process, and this short pilot study showed no observable inflammation or symptoms of foreign body reaction. D. Dziuba et al. [76] conducted long-term in vivo studies of ZEK100 and has reported conflicting in vivo results where there were pathological effects observed on the host tissue, despite achieving good in vitro biocompatibility results.

**Table 3:** In vivo test results for some of the biodegradable alloys as found in literature.

Alloy	Composition	Processing History	Comments on the Results	Animal	Location	Duration	Type of Implant	Implant Dimensions	Ref.
ZEK100	Mg-0.96 wt.% Zn-0.21 wt.% Zr-0.3 wt.% RE	Gravity die casting followed by direct extrusion.	ZEK100 did not show good biocompatibility. Pathological effects on the host tissue during complete degradation. This comes in spite of the favorable initial degradation and biocompatibility results.	Rabbit	Intramedullary tibia	9 months, 12 months	Cylindrical implants	2.5 mm dia, 25 mm length	[76]
ZX50	Mg-5 wt.% Zn-0.25 wt.% Ca-0.15 wt.% Mn	Direct Chill Casted (DCC) followed by hot extrusion	Good tolerance is observed.	Rat (Sprague-Dawley)	Femoral bone	24 weeks and 36 weeks 25 weeks and 36 weeks	Cylindrical pins	1.6 mm dia, 8 mm length	[30]
WZ21	Mg-1 wt.% Zn-2 wt.% Y-0.25 wt.% Ca-0.15 wt.% Mn	Direct Chill Casted (DCC) followed by hot extrusion	WZ21 encourages bone formation and gives evidence of osteoinductivity and osteoconductivity around magnesium.						
LAE442	Mg-3.7 wt.% Li-3.62 wt.% Al-0.73 wt.% Ce-0.38 wt.% La-0.16 wt.% Nd-0.03 wt.% Pr	Die casting followed by hot extrusion	Moderate gas formation and inflammatory reaction observed. Clinical tolerance deemed slightly lower than the austenitic stainless steel used as reference. Good regulation of the Mg levels by the body is observed, but Al and RE detected in the kidney, liver, and spleen. Conclusion: Prospective suitability of the alloy, although study was inconclusive on the final biocompatibility of the alloy.	Sheep	Right tibia	24 weeks	Intramedullary Interlocked Nailing system (nails/screws)	9 mm/3.5 mm dia, 130 mm/15–40 mm length	[3]
HP Mg	99.99 wt.% Mg	Cast, hot extruded, Rolled and Heat Treated	Good osseointegration compared to commercial PLLA screw, resulting in fracture healing 8 weeks after operation with increased bone density and mineralization.	Rabbit	Left femoral condyle	24 weeks	Screws	Major dia: 2.7 mm, Core dia: 2.1 mm, length: 27 mm, Pitch: 1 mm	[4]
Mg0.8Ca	Mg0.8 wt.% Ca	Machined from Extruded bar stock	Well-tolerated generally, although showed signs of slight reddening near the wound, which disappeared by 14 days after implantation. Mild to moderate amounts of gas accumulation was observed throughout the 8-week period.	Rabbit	Lateral cortex of tibia (both legs)	2,4,6,8 weeks	Screws	Major dia: 4 mm, length: 6 mm. Thread length: 5 mm, Core dia: 3 mm, Pitch: 1 mm.	[77]
LAE442	Mg-4.26 wt.% Li-3.30 Al-1.03 Ce-0.46 La-0.27 Nd-0.09 Pr	Cast and Extruded.	Clinically acceptable. No signs of deformity leading to lameness, swelling, pain, or gas formation was observed. Mg was well-degraded by 99.76%, but even after 3.5 years, the RE was not regulated or excreted out of the body, and Al, while present, had a decreased presence although Li was not detected.	Rabbit (New Zealand White Rabbits)	Intramedullary cavity of tibia	9 months, 3.5 years	Cylindrical pins	Dia: 2.5 mm, Length: 25 mm.	[78,79]
AZ31	Mg-2.5–3.5 wt.% Al-0.6–1.4 wt.% Zn-0.2–1.0 wt.% Mn	Commercial bought and hot extruded.	ZJ41 > WKX41 > AZ31 in terms of both degradation rates and volume of H_2_ evolution. Histological study showed no significant toxic effects on kidney, spleen, liver, lung, intestine, skin, skull, heart, and brain within the period.	Athymic Nude Mouse	Subcutaneous pocket on the back.	1 month	Disc	5 mm dia, 1.4 mm thickness	[80]
ZJ41	Mg-4 wt.% Zn-1 wt.% Sr-0.5 wt.% Zr	Cast and hot extruded.							
WKX41	Mg-4 wt.% Y-1 wt.% Zr-0.6 wt.% Ca								
JDBM	Mg-2.1 Nd-0.21 Zn-0.5 Zr (0.009Mn-0.006Si-0.005Cu-0.002Fe as impurities)	Alloy billet is machined, extruded, rolled, annealed, drawn and annealed	Study confirmed the safe metabolization of Mg and Zn. No sign of continuous accumulation of Nd and Zr in the organs (brain, lung, heart, liver, spleen, and kidney), although after the 1-month period, a sharp increase was detected in the liver and spleen. Lower aggregation of inflammatory cells compared to 316 L SS stent after 14 days. Endothelial cell recovery completed by 28 days. Ca concentration and degradation products decreased overtime without calcification of the vessel.	Rabbit (New Zealand White Rabbits)	Common carotid artery	1,4,12 months. 20 months	Stent	3 mm dia, 16 mm length, Stent strut thickness: 150 μm	[70,71]
Mg-Zn-Sr	Mg-6 wt.% Zn-0.5 wt.%Sr	Mold cast, Solution Treated and hot extruded	Increased peri-tunnel bone mass 16 weeks after ACL reconstruction surgery. Release of metal ions during degradation helps to heal. While the release of gases was expected to cause voids, no such large accumulation of gases was observed, which has been attributed to the excretion of the gas to local tissue via diffusion owing to the buffering role played by the knee-joint space.	Rabbit (New Zealand White Rabbits)—Male	ACL (Femur and tibia)	16 weeks	Hollow interference screw	3 mm outer dia, 8 mm length	[81]
AZ91	Mg-9 wt.% Al-0.9 wt.% Zn-0.1 wt.% Si-0.2 wt.% Mn-0.002 wt.% Fe-0.0005 wt.% Ni	Extruded, T6 heat treated	The good in vitro antimicrobial property is not found in vivo, tested against A. baumanii	Long Evans Rats (male)	Humeral head	7 days	Rods	1.6 mm dia, 16 mm length	[82]
WE43	Mg-4Y-3RE-Zr	Cast ingot hot extruded and machined.	No allergic or systemic reactions or complications during healing were observed. However, near the implant, foreign body reactions were observed.	Rabbit (New Zealand White Rabbits)—Male	Right tibia	16 weeks (4-week intervals)	Screws (w/pads)	Screw head dia: 3 mm, thread dia: 1.5 mm, core dia: 1.1 mm, length: 3 mm, pad thickness: 1 mm.	[83]
Mg-Ag-Y	Mg-0.95 wt.% Ag-0.92 wt.% Y	Cast, Homogenized, Hot extruded, Wire Drawn w/annealing after every two passes.	No abnormal effects detected after 6 weeks in liver, heart, and lungs. More than double the bone volume compared with PMg was detected.	Rat (Sprague-Dawley)	Distal femoral metaphysis (perpendicular to axis)	6 weeks	Rods (Intramedullary Nails)	1 mm dia,	[61]

## 4. Biodegradation

Degradation characteristics of the alloys can be classified as early fast degradation or slow degradation [30]. Slow degradation leads to the conservation of the structural integrity of the implant [30]. Fast degradation, on the other hand, is usually detrimental to the tissue and bone cells as demonstrated by the evolution of gases and non-uniform degradation.

### 4.1. Different Aspects of Biocorrosion Tests

#### 4.1.1. Electrochemical Measurements

Electrochemical tests were performed by a three-electrode system where the counter or control rod is composed of graphite [84] or, in other cases, it is platinum [64], the reference electrode is a saturated calomel electrode (SCE) [62,84,85,86], and the alloy specimen is the working electrode. Simulated body fluid (SBF) has been used as the solution medium [69].

#### 4.1.2. Potentiodynamic Polarization (PDP)

Potentiodynamic Polarization tests are used as an indication of how fast the corrosion reaction is [87]. This is useful in determining the rate of the degradation of a specific Mg alloy under the conditions tested. Mg alloys used as implants undergo biodegradation, forming Mg(OH)_2_ as a corrosion product [88]. Polarization tests using the Rotating Disk Electrode (RDE) have been employed by some researchers [85]. PDP scan rates of 0.5 mV/s [84,86] and 1 mV/s [64,88] have been most commonly used, and the corrosion potential is obtained from the PDP curves of Potential (V) vs. Current density (A·cm^2^).

#### 4.1.3. Immersion Tests

Immersion tests are usually governed by the ASTM-G31-72 standard [62,84,85,86,88]. Immersion durations have commonly varied between 30 days [84] and 7 days or as low as a 24 h. Immersion tests have been conducted to observe the pH change during degradation [86] and are also performed to weigh the mass loss of the alloy during degradation from which the corrosion rate (CR) can be calculated as per the standard mentioned above.

The common media used for immersion tests have been a variety of Simulated Body Fluids (SBF), including Hank’s solution, Dulbecco’s Modified Eagle Medium (DMEM), and Kokubo solution with, at times, slight modifications in composition. Hank’s solution has been found to be widely used in research as a medium for testing the degradation rates of biodegradable Mg alloys. Electrochemical and immersion tests were performed in this medium [84]. In some cases, Dulbecco’s Modified Eagle Medium (DMEM), as well as with 10% Fetal Bovine Serum (FBS), has been used for conducting immersion tests [85].

The temperature of the corrosion media also affects the corrosion results [89], which is why those corrosion studies concerned with biodegradation tests are usually carried out at the normal average human body temperature of 37 °C [62,84,85,86].

#### 4.1.4. Hydrogen Evolution Rates Measurement

Some studies have used the method of measuring the hydrogen gas evolved during the degradation of Mg alloys as an indicator of its rate of degradation. The hydrogen evolution is usually measured in mL/cm^2^/day. It has been used to deduce the corrosion rate in an immersion test by assuming the equivalency of 1 mL of H_2_ evolution to 1 mg of Mg dissolution [69]. In this method, the temperature difference between the standard atmospheric temperature and the body temperature is ignored for this assumption. However, it has also been reported that it cannot be correlated to the production of Mg ions as the stoichiometry of the redox reaction that it undergoes and is not fully understood [90]. For this reason, the hydrogen evolution measurements have not been discussed much in this review as a quantitative measure of biocorrosion.

#### 4.1.5. Modes of Corrosion

The main modes of corrosion, as found in the literature, appear to be either (a) the galvanic corrosion of the precipitates along the grain boundaries leading to the erosion of the grains, (b) micro-galvanic corrosion due to galvanic coupling of the α-Mg matrix and any solutes leading to corrosion within the grain itself, and (c) a combination of both. It is in this first case that grain refinement gives the advantage of encouraging uniform degradation.

D. Liu et al. [91] have discussed the correlation between the grain size and corrosion resistance. For instance, the microstructure development resulting from multiple hot extrusions were observed to achieve better corrosion resistance. This has been further discussed in the heat treatment section.

#### 4.1.6. Effect of pH

Biocorrosion is more complex than simply considering the effects of a stagnant corrosive media alone. In reality, the biocorrosion environment to which the implants would be exposed depends a great deal on the metabolic activities of the surrounding tissues and their absorption rates of the degradation products. This, in turn, means that the surrounding cell types need consideration when testing for the application/site specific degradation rates. A. Witecka et al. [92] studied the corrosion behavior of ZM21 in the presence of SaOS_2_ cells and has observed increased degradation, which has been attributed to the decrease in pH of the medium due to the metabolic activities of the cells.

## 5. Effect of Individual Alloying Element on Mg Alloy Biodegradation

### 5.1. Magnesium

A wide range of data are available on the corrosion characteristics of Mg due to degradation tests performed on Pure Mg as a reference in virtually all the corrosion tests conducted in this field. The test results usually vary from one to the next due to the difference in testing parameters, work history, and, more importantly, due to differences in the purity of the Mg. The duration of the test also affects the results due to the change in corrosion rate over time, otherwise known as corrosion characteristics. J. Hoffstetter et al. [93] conducted an immersion test in NaHCO_3_/CO_2_-buffered SBF and determined the degradation characteristics on the basis of the hydrogen evolution rates for high purity (HP) and ultra-high purity (XHP) Mg. The study determined that the as-cast XHP Mg had an average degradation rate of ~10 μm/year and the HP Mg degraded at ~28 μm/year while the HP Mg, after annealing, degraded at ~39 μm/year. Though these rates are deductions based on the hydrogen evolution rate, they are mentioned as a qualitative assessment. It shows that both the purity and the work history affect the degradation rates of the Mg. In direct studies involving the use of Hank’s solution, corrosion rates between 0.22 mm/year (as-rolled) amd 0.36 mm/year (as-cast) have been obtained for the same duration of study (500 h) [62]. Meanwhile, the weight loss method using DMEM + 10% FBS has yielded as low as 0.66 ± 0.36 mm/year for 168 h [65].

### 5.2. Iron

G. Xie et al. [94] reported that, in a binary Mg-Fe alloy, specifically the Mg_30_Fe_70_ alloy, alloying with Fe decreased the degradation rate significantly. However, Fe, which is usually present as impurities in many of the Mg alloys, has been said to cause an increase in degradation.

### 5.3. Calcium

An increase in Ca content in as-cast Mg-Ca alloys have been observed to result in increased corrosion rates [95]. It has been reported by R.-C. Zeng et al. [96] that the effect of Ca on the biodegradation of binary Mg-Ca alloys is dual in nature. As a grain refiner, it decreases the corrosion rate of the alloy, but at the same time, it accelerates the corrosion rate due to galvanic coupling between the Mg_2_Ca phase and the α-Mg matrix. However, modifying this secondary phase has been reported to result in decreased corrosion rates compared with pure Magnesium [97].

### 5.4. Zinc

The Zn effect on Mg degradation is dual in nature. Zn added to form Mg-6Zn reduced the corrosion rate due to grain refinement leading to a more uniform corrosion surface [98]. This could also be due to the solid solution treatment and hot working performed on the alloy leading to a uniform single phase. Similarly, Mg^2+^ dissolution into Hank’s solution has been reportedly reduced in a Mg6.5 wt.%Zn alloy produced by mechanical milling [99]. However, when tested in SBF, the addition of Zn has been reported to lead to decreased corrosion resistance [100]. The increase in corrosion rate with the increase in Zn content, as immersion tested in 3.5 wt.%NaCl solution, has also been reported in the literature [101]. Y. Yan et al. [102] reported that an increase in Zn content achieved a reduction of corrosion potential, albeit an increased corrosion current owing to microgalvanic corrosion resulting from the increased sizes of Mg-Zn intermetallics. So, the overall effect of Zn on Mg corrosion would depend on the work history and the design of the implant in addition to the specific environment it is exposed to.

### 5.5. Copper

The addition of Cu to Mg results in increased degradation rates, which increases along with the Cu content [65]. This has been attributed to the presence of Mg_2_Cu precipitates acting as a cathode in the galvanic couple formed between the Mg matrix and the secondary phase. Binary Mg-Cu alloys have shown to increase pH in in vitro studies, which is expected to inhibit bacterial growth. Though in vivo, it is thought that the homeostatic regulation of the pH would take place at the site of implant.

### 5.6. Silicon

Si has been reported to decrease the corrosion potential of binary Mg1Si alloy compared to unalloyed Mg [62]. Although the corrosion rate of as-cast alloys increased significantly, after rolling, it achieved lower corrosion rates than unalloyed Mg. This change in corrosion rate after working could be the result of the modification of the secondary-phase Mg_2_Si, which is formed when alloyed with Mg [103], the presence of which provides cathodic corrosion initiation sites [104].

### 5.7. Tin

J. Kubásek et al. [63] has reported that in binary Mg-Sn alloys, Sn improves the degradation rate when existing as less than 1 wt.% concentrations, while worsening the degradation rate when present in higher concentrations due to the galvanic effect of secondary-phase Mg_2_Sn. Increasing the presence of the Mg_2_Sn intermetallic while promoting passivity also acted as initiation sites for pitting corrosion, as has been reported by H.-Y. Ha et al. [105]. They also noted an increase in the H_2_ gas evolution rate accompanied with the increased secondary phase, while at the same time, Sn dissolved in the matrix was noted to reduce the H_2_ gas evolution rate.

### 5.8. Strontium

**Figure 4:**
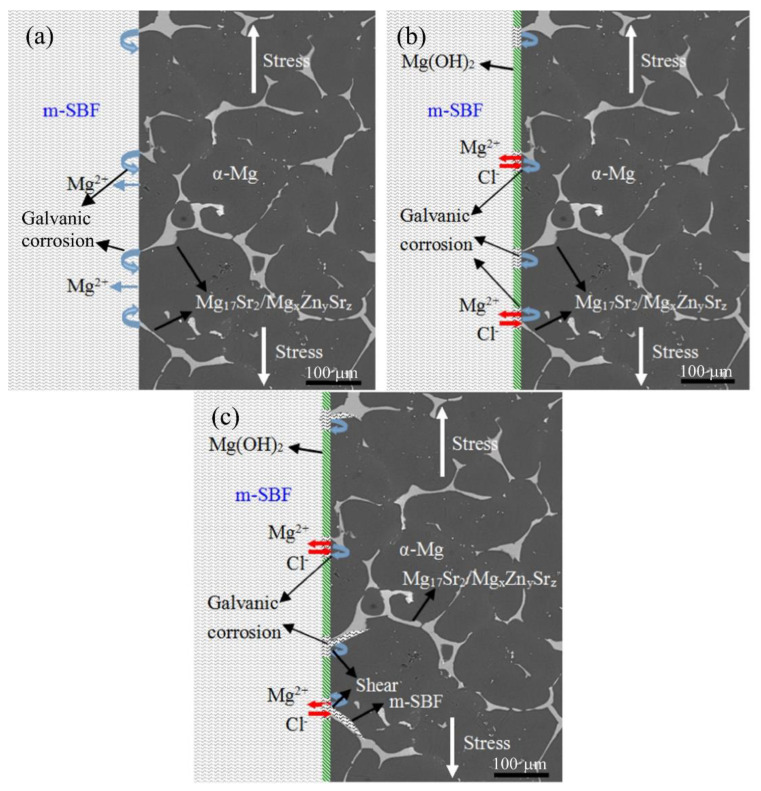
Schematic diagrams for intergranular stress corrosion cracking (IGSCC) propagation in m-SBF: (**a**) Galvanic corrosion of Mg matrix with grain boundaries; (**b**) galvanic corrosion developed through the partially Mg(OH)_2_ film; (**c**) accelerated galvanic corrosion along the grain boundaries under stress [107].

An Sr content of up to 2 wt.% has been observed to produce decreased corrosion rates in hot-rolled binary Mg-Sr alloys [88]. A further increase in Sr content reportedly increased corrosion rates of the alloys. M. Bornapour et al. [106] reported that, in the binary alloy of as-cast Mg-0.5Sr, heat treatment had resulted in significantly increased rates of degradation in SBF. L. Chen et al. [107] reported that there is an increase in susceptibility to IGSCC with an increase in Sr from 0.4 wt.% to 1.6 wt.% in ZK40 alloys. This was attributed to the increase in micro-galvanic corrosion between the grain boundary precipitates and the α-Mg as shown in Figure 4. Similar results of increasing Sr content leading to increased corrosion rates have also been reported by C. Zhao et al. [108]. However, X.N. Gu et al. [88] reported a decrease in the corrosion rate of binary Mg-xSr (x: 1–4 wt.%) alloys produced via casting and subsequent multi-pass rolling when compared with pure Mg. The study of J. Han et al. [109] also mentioned the contribution of the intergranular precipitates to the micro-galvanic corrosion and further noted that, by extruding the alloy, it resulted in slower degradation rates, which have been attributed to the breaking and redistribution of the Mg_17_Sr_2_ secondary phase. Homogenization treatment of Mg-Sr alloys at 450 °C for 12 h followed by water quenching have also been reported to reduce the quantity of the secondary-phase Mg_17_Sr_2_ and achieve reduced corrosion rates [110].

### 5.9. Manganese

Decreased corrosion resistance with the addition of Mn to Mg has been reported in a binary alloy of Mg-Mn, tested both in Hank’s solution and SBF [62]. However, D. H. Cho et al. [111] has observed that, in tests, conducted on Mg4Zn0.4Ca in Hank’s solution, the addition of Mn resulted in a decrease in the depth of the corrosion product attributed to the formation of a Mn oxide film providing protection against the attack of chloride ions.

### 5.10. Aluminum

Aluminum contributes to corrosion inhibition by providing a passivating film. Most of the literature on the effect of Al concerns the effect of secondary phases formed in the presence of other alloying elements, and as such, the sole contribution of Al to Mg degradation is somewhat unclear. X. Gu et al. [62] concluded that, overall, the addition of Al decreases the corrosion rate of an as-cast binary Mg-1Al alloy [62] both in SBF and Hank’s solution compared to unalloyed pure Mg. The hydrogen evolution rate of Mg-1Al was lower in both SBF and Hank’s solution of as-cast alloys. When the alloys were tested after rolling, it was found to have accelerated H_2_ evolution rates [62]. However, the reported electrochemical data suggests otherwise, albeit the decrease in corrosion performance of as-cast alloy is slight compared to unalloyed Mg. The tests conducted on TZ81 alloys (Mg-8 wt.%Sn-1 wt.%Zn) have demonstrated that Al forms an intermetallic phase of Al_5_Fe_2_ with the impurity Fe content of the alloy, and between 1 and 3 wt.% of Al has been reported to lead to increased corrosion rates, though at 0.3 wt.% Al, it has been reported to have slightly reduced corrosion rates [112].

### 5.11. Bismuth

It has been reported by S. Remennik et al. [113] that Mg-5Bi-1Si and Mg-5Bi-1Ca (all in wt.%) alloys produced via Rapid Solidification followed by extrusion showed rapid degradation, although the Mg-Bi-Ca alloy have been noted for their lack of observable gas formation in rabbit femur implants. More recently, H. Y. Tok et al. [114] has also reported its grain refinement with increasing Bi content in Mg-1.2 wt.%Ca. The secondary-phase Mg_3_Bi_2_ forms below 3 wt.% of Bi, and Mg_2_Bi_2_Ca forms as precipitates above 5 wt.% of Bi [114]. Their conclusion was that the secondary-phase Mg_2_Bi_2_Ca formed as precipitates contributed to the increased corrosion rate by galvanic coupling with the α-Mg matrix. However, with 0.5 wt.% Bi, the corrosion rate was reduced, attributed to its presence inside the Mg matrix as a solute due to the low Bi concentration. Despite these conclusions, the results of their study did not indicate any significant decrease degradation rates compared with Mg-1.2 wt.%Ca, i.e., with no Bi alloyed.

### 5.12. Scandium

T. Li et al. [115] reported that the addition of 0.2 wt.% Sc to ZK21 (ZK21-0.2Sc) resulted in reduced hydrogen evolution and decreased degradation rates compared with pure Mg and ZK21.

### 5.13. Gallium

Gallium has been reported to lower the corrosion rate at low concentrations (below 1 wt.%) while it increased the degradation at higher content when studied in binary Mg-Ga alloys [63]. The secondary phase, Mg_5_Ga_2_, acts as the cathode compared to Mg, and galvanic corrosion takes place at the vicinity of this secondary phase.

**Figure 5:**
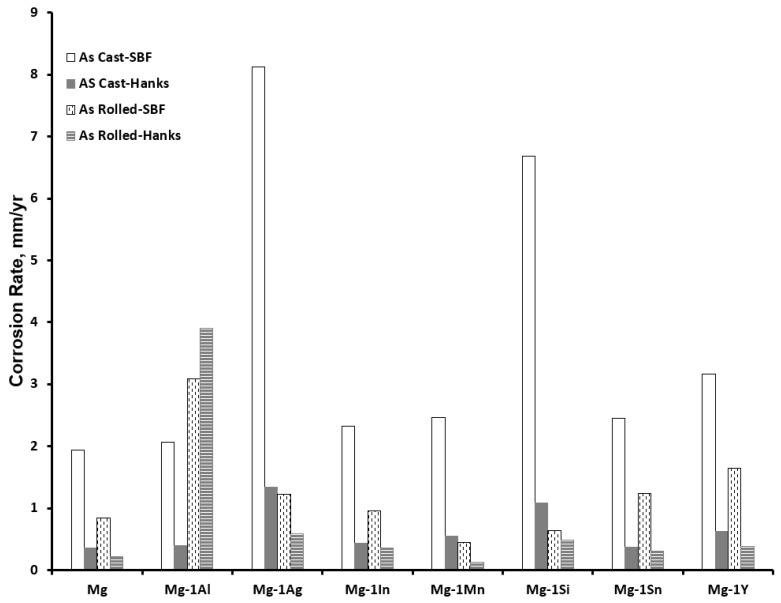
Effect of work history on the degradation rates of binary alloys of biodegradable Mg alloys in SBF and Hank’s solution for the immersion time of 500 h [62].

Table 4 below shows the summary of the elemental effects on the degradation rates of Mg alloys as found in the literature. The summarized results are based on the reported effect of the element on the Mg alloys as discussed above and considers the various work histories through which the alloys were produced and the different test durations of the corrosion tests. These differing factors would explain the differing corrosion rates that have been reported for each element. Figure 5 presents the effect of work history on the degradation rates of binary alloys of biodegradable Mg alloys in SBF and Hanks solution for an immersion time of 500 h. It can be observed that the corrosion rates of all the alloys decreases upon hot-rolling except for the Mg-1Al alloy. The accelerated corrosion rate is attributed to the precipitation of a eutectic α phase during hot-rolling [62]. Among all the Mg-1X alloys, the as-rolled Mg-1Mn alloy exhibited not only the lowest corrosion rate but also a lower corrosion rate compared to as-rolled pure Mg in both SBF and Hank’s solution.

## 6. Microstructure and Mechanical Properties Mg Alloys

### 6.1. Effect of the Alloying Elements

The microstructures of Mg-alloys are mainly composed of an α-Mg matrix with some amount of alloying element in them, followed by secondary phases. These secondary phases are primarily located in the grain boundaries. The mode of formation when casting is that the secondary phases accumulate in advance of the forming grains. The secondary phases appear as precipitates along the grain boundary [88]. However, in some cases, the secondary phases are also present in the dendritic structures, if present [110].

The microstructure of the Mg alloy is dependent on the alloying elements as well as the work history of the alloy. This can be seen from the results of Table 5.

#### 6.1.1. Mg

Mg in its pure form exists in an α-Mg phase, which has hexagonal close pack (HCP) structures with dimensions of a = 0.32 nm and c = 10.3 nm [88]. The addition of further alloying elements render the different characteristics associated with the formation of their respective secondary and tertiary phases. These initially formed phases undergo additional changes during further processing of the Mg alloy. Therefore, it is necessary to look at some of the alloying elements and the commonly formed phases with Mg found in the literature. For this purpose, only the elements with sufficient biocompatibility have been chosen for further discussion. Pure Mg has an ultimate compressive strength (UCS) of approximately 185.67 MPa and an ultimate tensile strength (UTS) of approximately 63 MPa [65].

#### 6.1.2. Zn

Zinc forms the intermetallic phase MgZn, which is mainly present in the grain boundary [116]. It has a solubility limit of approximately 2.6 wt.% in Mg [117]. It has been reported by C.J. Boehlert and K. Knittel [118] that Zn of 4 wt.% produced the highest refinement of the grain size in the Mg binary alloy. Similarly, S. Cai et al. [116] indicated that the addition of Zn up to 5 wt.%. increased the mechanical properties of Mg alloys, which has been attributed to the grain refinement, solid solution strengthening, and second-phase strengthening. Based on this, optimal Zn content can be said to be between 4 and 5 wt.% for grain refinement. On the other hand, the elongation % was the highest when 1 wt.%. Zn was used [116].

Mg-Zn alloy produced from powder metallurgy produced fine, equiaxed grains and row elongated grains. Strike-like coarse intermetallic phases were also reportedly produced with increased Zn concentrations [119]. Zn addition to Mg-6 wt.%Sn was investigated by N. El Mahallawy et al. [120] with the addition of Zn wt.% of 2 and 4. In as-cast alloys, Zn acted as a grain refiner to the Sn, further complimenting the grain-refining characteristic of Sn itself. Zn addition increased the grain size of as-rolled and as-extruded alloys as well.

#### 6.1.3. Ca

Y. C. Lee et al. [121] reported that the addition of Ca up to 0.4 wt.% resulted in significant grain refinement of approximately 270 μm, and any additional grain refinement was reported to have only minor changes in grain size. Research conducted by Z. Li et al. [64] on Mg-Ca binary alloys with 1–3 wt.% of Ca found that the yield strength (YS), UTS, and elongation of the binary Mg alloy decreased with increasing Ca content for the as-cast alloys. The UTS and elongation were successively increased after hot rolling and hot extrusion [64]. The loss in mechanical properties has been attributed to the embrittlement of the alloy owing to the secondary phase Mg_2_Ca. An increase in this Mg_2_Ca was found to enhance the corrosion rate of the alloy. The formation of the Mg_2_Ca phase, in proximity to Fe and Si, have been reported to lead to pitting corrosion [96].

Meanwhile, H.R.B. Rad et al. reported that increasing the Ca content from 0.5 wt.% to 10 wt.% significantly increased the hardness of binary Mg-Ca alloys [86]. Mg-0.79Ca with its high hardness, UTS, YS, and corrosion resistance was found to be the most promising Mg-Ca composition for use as a biodegradable material by R.-C. Zeng et al. [96]. In the presence of Si, the CaMgSi phase is present in higher-order alloys [122].

#### 6.1.4. Cu

Recent works by C. Liu et al. [65] produced Mg-Cu alloys of approximate grain size 100 μm. Mg_2_Cu secondary-phase precipitates form with an increasing Cu content (0.05, 0.1, 0.5 wt.%.) and have been reported to be present as a discontinuous distribution along the grain boundaries as well as in the grains as particles [65]. A similar study by Y. Li et al. [123] in their supplementary data reported obtaining a Mg-Cu alloy grain size of approximately 300 μm, and with an increasing Cu content (0.05, 0.1, 0.25 wt.%.), the presence of secondary-phase Mg_2_Cu becomes more visible in the form of a globular presence, mainly at the grain boundaries and a small amount inside the grains.

The presence of the secondary-phase Mg_2_Cu has also been credited with better mechanical properties of the Mg-Cu alloys compared with pure Mg [65]. Mg-0.03Cu has been reported to have a UCS of 199.67 MPa and a UTS of approximately 83 MPa, with these values decreasing with the increase in Cu content [65].

Cu has also been added to Mg-Zn alloys to enhance the mechanical properties, which gives rise to the intermetallic phases of Mg(Zn,Cu) and Mg(Zn,Cu)_2_ [124]. M. Lotfpour et al. [124] reported that when added to Mg-2 wt.%Zn, there is an increase in mechanical properties of UTS and ductility until approximately 0.5 wt.% Cu, after which it decreases drastically, even well below that of the original alloy. This behavior has been attributed to the embrittlement of the alloy due to an increase in cleavage planes resulting in brittle fracture [124].

#### 6.1.5. Si

Si is also a grain refiner of Mg [121]. Si has been reported to have produced approximately 240 μm. Si forms the secondary phase of Mg_2_Si, which, after annealing, becomes finer and more homogenized [122]. If the Si concentration becomes more than the eutectic concentration limits, that secondary phase crystallizes in the form of needles, resulting in increased brittleness [125]. Si, when added to Mg alloys containing Ca, forms CaMgSi [122].

#### 6.1.6. Mn

The maximum solubility of manganese in magnesium is only approximately 2.2 wt.% [126]. It has been reported by Gu et al. [62] that the addition of Mn below this volume results in complete solubility of Mn in Mg and only a purely α-Mg matrix is formed. Moreover, they also noted that the addition of Mn does not contribute to an enhancement of strength, but rather lowers the elongation.

#### 6.1.7. Sn

Tin has a solid solubility limit of 14.5 wt.% in Mg [127] and forms the secondary-phase of Mg_2_Sn in binary alloys [63,67,128], which may not be detected by XRD in low volumes (<3 wt.%) [67]. This eutectic phase is found as particles between the α-Mg dendrites [63]. C. Zhao et al. [67] reported that with 1 wt.% Sn, a near equiaxed grain structure was formed while Sn content of 3 wt.% and higher gave rise to dendrites of α-Mg where the secondary dendrite arm spacing of the alloys decreased with increasing Sn content. Binary alloys of Mg-Sn are composed of an α-Mg matrix, a eutectic composition of α-Mg + Mg_2_Sn, and Mg_2_Sn in a devoiced manner, as well as a distribution of tiny white particles [128]. For 5 wt.% Sn, UTS of over 130 MPa (40.7% increase), and an elongation of approximately 120 MPa (39.3% increase) compared with pure Mg, has been reported by H. Liu et al. [128]. Further increase in Sn content reduces both the UTS and ductility.

#### 6.1.8. Al

Al has a maximum solid solubility of 13 wt.% in Mg at eutectic temperatures [129]. Though the eutectic phase should theoretically appear at approximately 13 wt.% Al, it is present in as low as 2 wt.% Al during non-equilibrium cooling processes such as casting [129]. A β-Mg_17_Al_12_ phase forms in the grain boundaries and inter-dendritic regions of the alloy, with the dendrites being α-Mg. Additionally, the eutectic α-Mg with high concentrations of Al is also expected to be found in grain boundaries [129]. It has been reported that the addition of Al increases the porosity of the Mg alloy, until approximately 11% wt of Al is reached, after which the porosity decreases [conference paper]. The increase in Al content also correlated with an increase in the pore size of the alloy. Additionally of note is the undesirable interaction between zirconium and aluminum, which seems to limit the use of Zr along with Al in Mg alloys [129].

#### 6.1.9. Sr

Sr is known as a grain refiner of Magnesium alloys whereby it has been reported that an increase in its wt.% of 0.5–2% decreased the average grain size of binary Mg-Sr alloys [110]. Sr has a maximum solid solubility of 0.11 wt.% at a eutectic temperature [110] and its secondary phase with Mg is present as Mg_17_Sr_2_ [122] but is mostly concentrated in the grain boundaries [110]. These Mg_17_Sr_2_ have been reportedly been present in binary alloys of Mg-Sr (with wt.% 1–4) in a hexagonal structure where the base sides measure 10.469 nm with a c value of 10.3 nm [88]. For binary alloys of Mg-Sr, an increase in Sr wt.% of up to 2% has been found to increase the TYS and UTS [88]. H. Liu et al. [130] reported that the addition of Sr to the as-cast alloy Mg-5 wt.%Sn refined the microstructure and produced rod-shaped and bone-shaped secondary-phase MgSnSr, and the optimum mechanical properties were achieved with 2.14 wt.% Sr content. Additional Sr content resulted in decreased UTS and elongation, although TYS increased.

#### 6.1.10. Zr

This is normally added as a grain refiner, though its use in Mg alloys containing Al is not advised due to undesirable interactions between Zr and Al [129].

#### 6.1.11. Bi

Bismuth is known to be a grain refiner of Mg alloys [113,114,131]. Increasing Bi increased the grain refinement. For Bi up to 3 wt.%, a Mg_3_Bi_2_ phase was formed, and when used alongside Ca, Mg_2_Ca was formed, while between 5 and 12 wt.%, a Mg_2_Bi_2_Ca phase was present along with the Mg_3_Bi_2_ [114]. Greater than 0.5 wt.% of Bi has been attributed to a greater role of galvanic corrosion between the primary α-Mg and the secondary phases [114].

**Figure 6:**
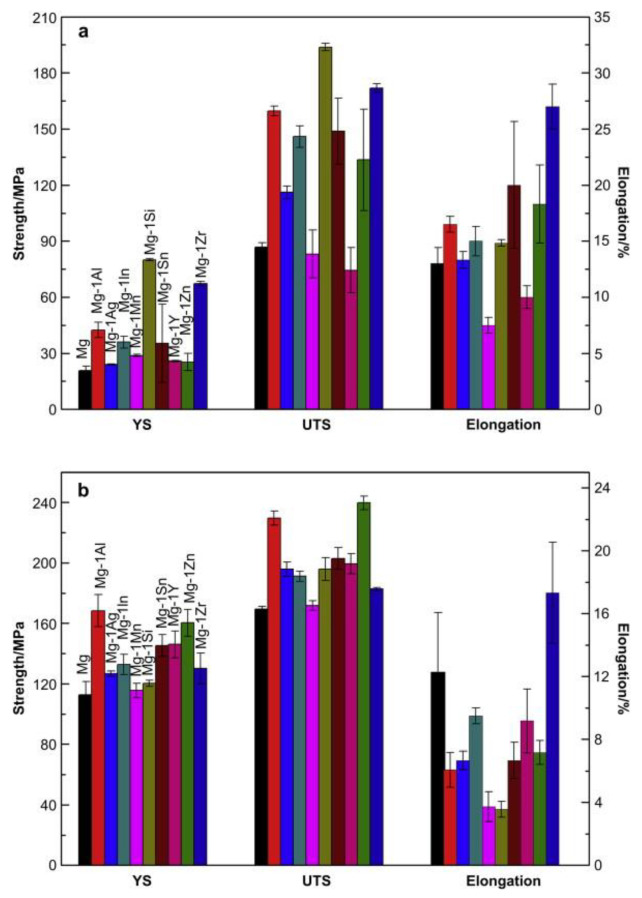
Tensile properties of (**a**) as-cast and (**b**) as-rolled pure Mg and Mg–1X alloy (X = Al, Ag, In, Mn, Si, Sn, Y, Zn, and Zr) samples at room temperature [62].

Table 5 and Figure 6 presents the effect of alloying elements on tensile properties of Mg-1X alloys in both as-cast and as-rolled conditions. While the addition of the alloying elements has improved the tensile properties, the addition of Mn and Y has reduced UTS and the percentage elongation of the alloy in the as-cast condition. On one hand, the addition of alloying elements has improved YS and UTS of all the as-rolled Mg-1X alloys, but on the other, has reduced percentage elongation except for Zr. Hot-rolling significantly improved the strength properties of as-cast Mg-1X alloys with an obvious reduction in percentage elongation.

#### 6.1.12. Sc

0.2 wt.% Scandium added to ZK21 [115] has been reported to result in the grain refinement of ZK21.The alloy was as-cast and the resultant XRD analysis showed a single primary phase similar to the pure Mg pattern, indicating an α-Mg matrix with dissolved alloying elements.

### 6.2. Effect of Processing

#### 6.2.1. Liquid Metallurgy

Liquid metallurgy has the major advantage of producing bulk alloys and producing the starting material for various alloys. It is a principal process through which Mg alloy billets are produced for further processing. It has the chief advantage of being economically scalable for the large-scale industrial output of Mg alloys. All the alloys mentioned in this paper, except that which has been produced by Powder Metallurgy (PM) or the newly emerging field of Additive Manufacturing (AM), have been produced by any one of the various casting processes.

O. Hakimi et al. [132] have reported an improvement in the resistance to SCC (Stress Corrosion Cracking) of the Mg alloy EW62 (Mg-6Nd-2Y-0.5Zr, all in wt.%) produced by the RS (Rapid Solidification) process followed by extrusion.

#### 6.2.2. Powder Metallurgy

In recent years, a number of researchers [133,134,135,136,137,138,139] that produced Mg alloys used the Powder Metallurgy process route. This process is usually followed by extrusion to form the final alloy. PM has the advantage of uniform dispersal of the elements, and thereby uniformity of the secondary phases and a greater dissolution of the alloy elements in the αMg matrix.

J. Kubásek et al. [134] reported the superior properties of TYS and hardness of the WE43 (Mg-4Y-3RE-Zr) alloy produced via extrusion of the alloy prepared by powder metallurgy when compared with that of the extruded alloy prepared using casting. In particular, the effects of solid-solution strengthening and precipitate strengthening achieved as a result of the more uniform distribution of elements via the PM method has been noted. The ability to produce a uniform distribution of the secondary phases has also been reported by M. Rashad et al. [135] using the conventional PM route followed by hot extrusion. Grain size refinement of as much as 500 nm has been reported for Mg-6Zn-5Ca produced via PM [137].

#### 6.2.3. Extrusion and Rolling

Secondary mechanical processing are commonly used in Mg alloys to improve mechanical properties by modification of microstructure. In the study conducted by N. El Mahallawy et al. [120] on Mg-6Sn-xZn alloys (x: 0, 2, 4 wt.%), it was determined that grain sizes were vastly refined after extrusion and after rolling when compared with as-cast alloys [120], with the as-rolled alloys having the most refinement. Furthermore, the results of mechanical testing determined that the highest YS and UTS were produced for extruded alloys, followed by rolled alloys, with each of the processes producing superior strength for all values of Zn [120]. J. Su et al. [140] reported that a higher rolling speed (1000 m/min) achieved better rollability and weaker texture of the AZ31 alloys compared to the slower speed of 15 m/min. These results have been attributed to the dynamic recrystallization taking place owing to the increased temperature of the alloy and the activation of a larger number of twinning and slip systems—in particular, the <c + a> pyramidal slip system.

However, in the case of the extrusion process, researchers have used both the direct extrusion process [141,142] and the indirect extrusion process [105,143,144] to process the Mg alloys. Extrusion has the advantage of grain refinement of the Mg alloy via plastic deformation, and in the case of the alloy being produced via the Powder Metallurgy route, it also offers the advantage of increasing the bonding between the sinter-bonded particles, i.e., decreasing the porosity of the alloy.

#### 6.2.4. Equal Channel Angular Pressing/Extrusion (ECAP/ECAE)

**Figure 7:**
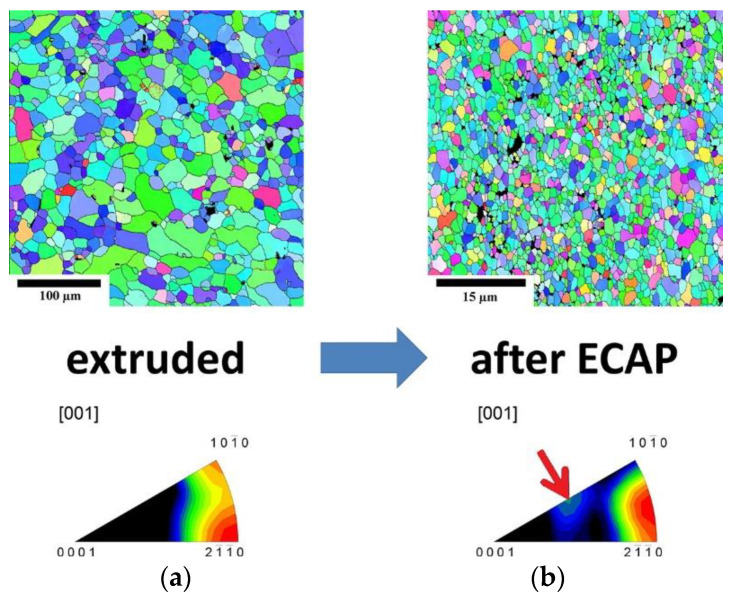
Electron back-scattered diffraction orientation maps of LAE442 (**a**) after extrusion and (**b**) after ECAP, with orientation triangles [146].

ECAP or ECAE [145], on the other hand, has the added advantage of producing grain refinement as well as equiaxed grains in comparison with extrusion via the change in direction of plastic deformation of the alloy. Through work carried out on LAE442 by P. Minárik et al. [146], it was found that the grain size of ~1 mm obtained by casting was refined down to ~1.7 μm after hot extrusion (350 °C) at a ratio of 22:1 followed by 12 passes of ECAP at 90° (See Figure 7). Similarly, P. Minárik et al. [147] reported achieving higher degradation resistance using the ECAP process for LAE442 as compared with extruded LAE442 with no adverse effects in cytocompatibility noticed due to the additional ECAP process. Y. Tan et al. [148] conducted multiple passes of ECAP on a Mg–2Y–0.6Nd–0.6Zr alloy at different temperatures and reported improved tensile strength of the material.

### 6.3. Effect of Post-Processing Treatment

The heat treatment and surface modification of the Mg alloys are two of the main post-alloy processing methods found in the literature.

#### 6.3.1. Effect of Heat Treatment

D. Liu et al. [91] attributed the grain refinement and solid solution strengthening to improved mechanical properties of the extruded alloy compared to the as-cast alloy.

It has been reported that the age-hardening response increases with increasing Sr content [110]. Homogenization of as-cast Mg-xSr (x: 0.5, 1, 2 wt.%) at 450 °C for 12 h followed by quenching in water dissolved the dendritic structures of the as-cast Mg-Sr alloy but it had no effect on the grain size [110]. Aging at 160 °C for 30 to 300 h showed that there was a reduction in hardness though it increased the UTS, TYS, and CYS [110]. H. Ibrahim et al. [149] performed solution treatment (510 °C, 3 h) age hardening in an oil bath (200 °C; 1 to 10 h) of a cast Mg alloy (Mg-1.2 wt.% Zn-0.5 wt.% Ca) and found that the heat treatment improved both the mechanical and degradation properties of the alloy. As an example of thermomechanical processing, H. B. Henderson et al. [150] recently reported an improvement in both the mechanical and corrosion properties of an as-cast Mg-1 wt.%Ca-0.5 wt.%Sr alloy billet by hot extrusion (275, 340, and 400 °C) at a ratio of 25:1. Extrusion served the purpose of providing the large driving force required to break the structure of the interconnected as-cast eutectic phase, as well as bringing about further grain refinement resulting from the severe plastic deformation it undergoes. Grain sizes as low as 1.6–6.3 μm were obtained at an extrusion temperature of 275 °C. Moreover, the process lowered the degradation rate while at the same time showed low toxicity for mouse osteoblasts. On the other hand, it also improved the maximum tensile yield strength up to 304 MPa, which is even higher than annealed 316L SS.

D.-J. Lin et al. [151] used both the solid solution treatment (345 °C, 10 h) and a strain-induced melting activation (SIMA) heat treatment (355 °C and 370 °C) on the extruded ZAX1330 alloy and also noted the role of grain refinement in improving the mechanical strength and corrosion resistance. Heating the alloys decreased the elastic modulus. Improved UTS and elongation, despite decreased YS, following the T4 treatment was observed when compared with the as-extruded alloy. Heat treatment up to 355 °C resulted in a uniform distribution of the secondary phases, whereas higher heating produced segregated regions of secondary phases due to the matrix melting at 370 °C and the solid-solution Ca diffusion into the liquid phase [151]. T4 produced the lowest corrosion density due to the near elimination of Ca_2_Mg_6_Zn_3_ precipitates by the solid solution effect of Ca dissolving into the matrix, leading to the dispersion of Mg-Zn precipitates.

An example of the corrosion mechanism being altered due to heat treatment is found in the case of a MgAlGd alloy, whose main corrosion mechanism of as-cast and T4-treated alloys changed from filiform and intergranular corrosion to that of needle-like precipitates within the grains, acting as a barrier to corrosion of the matrix [152].

#### 6.3.2. Surface Modifications

The development of Mg alloys is many times complemented by the development of various surface modification characteristics of the alloys/implants. The research in these areas concern the alteration of the implant surface characteristics in order to affect the corrosion characteristics as well as improve the initial cell adhesion and proliferation characteristics. These surface alterations are mainly of either the application of a coating on the alloy/composite surface or modification of the implant surface properties by various means. Both these methods impart different physical characteristics of the surface. A solution to develop biodegradable Mg alloy implants according to the different physical environments required by the various applications and locations could lie in tailoring application-specific surface modifications on high-performance Mg alloys.

##### Surface Coating

One of the most common coating materials have been Hydroxyapatite (HA) [153]. The use of Micro Arc Oxidation techniques (MAO) for surface coating are also commonly found in the literature [153,154,155,156]. Tang et al. used this method to coat AZ31 with HA (Hydroxyapatite) and reported that it has induced additional resistance of the alloy to corrosion in Simulated Body Fluid by the barrier effect and the degradation of the coating itself. HA coating has also been said to result in enhanced osteoblast development compared to uncoated Mg alloy samples along with a significant reduction in the rate of degradation [157]. Furthermore, N. Yu et al. [158] reported that doping Strontium into the HA coatings to produce a dual layer of SrHA by microwave irradiationled to increased corrosion resistance of the initial stages of the Mg alloy. They also worked on commercially available AZ31 alloy.

Others such as H.R. Baksheshi-Rad et al. [159] have coated Mg-1.2Ca-2Zn samples with a coating of HA and ostacalcium phosphate (OCP) followed by a layer of polycaprolactone (PCL) and reported a significant reduction in corrosion current densities as compared with uncoated samples. They used a combination of chemical solution deposition and the dip coating method to apply the coating on the samples. Su et al. [160] fabricated a composite coating of calcium phosphate (CaP) and collagen (Col) on the surface of AZ60 Mg alloy by chemical conversion and dip-coating methods. The composite coating not only reduced the in vitro degradation rate of Mg alloy effectively because the collagen coating sealed the pores in the CaP coating but also improved the biocompatibility via effective promotion of cell adhesion and proliferation. Li et al. [161] applied a multilayered coating on a Mg substrate. The coating contained a chemical conversion layer of fluoride or phosphate, an adhesion layer, and a layer of biodegradable polylactic acid (PLA). The corrosion rate of the coated Mg has been reduced to one-tenth that of the uncoated bare Mg.

One-step electrodeposition of a Ce-based hydrophobic surface on Mg alloy was carried out by Yan liu et al. [162]. S. Shen et al. [157] used a technique known as the Rapid microwave aqueous chemical route to produce an HA bilayer on Mg alloy by microwave irradiation. The use of the electrospinning method to coat AZ31 with composite of Polycaprolactone (PCL) and ZnO nano particles of 1 wt.% and 3 wt.%, used by J. Kim et al. [163], produced increased biocompatibility and corrosion resistance. P. Tian et al. [164] combined the use of plasma electrolytic oxidization (PEO) and Hydrothermal treatment to apply a coating of HA using the latter on the coating applied using the former. The additional secondary treatment has been attributed to the improved surface biocompatibility via improved cell adhesion and proliferation.

J. Tang et al. [165] attempted a comparison between micro arc oxidation (MAO) and electrophoresis deposition (EPD) methods. Though both coating methods provided corrosion resistance for the Mg-Zr pins used, the study was not performed on the same coating components and the difference in reported results may have been due to a component factor rather than a definitive statement of the processes used. Deposition of a CaP (Calcium Phosphate) ceramic coating on Pure Mg, Mg-0.6 wt.%Ca and Mg-0.55 wt.%Ca-1.74 wt.%Zn alloys by MAO was reported to yield a better corrosion protection in SBF, though in Tris-HCl immersion, it had the opposite effect of increased corrosion compared with uncoated alloys [166]. Furthermore, Razavi et al. [167] deposited a nanocomposite coating made of diopside, bredigite, and fluoridated hydroxyapatite bioceramics on a micro-arc oxidation-treated surface of a biodegradable AZ91 Mg alloy by the electrophoretic deposition method. Improved corrosion resistance and implant osteointegration were reported to be achieved. X. Yu et al. [168] reported on improved corrosion resistance achieved by oxidizing Y alloys of Mg, i.e., Mg-1.5 wt.%Y and Mg-1.5 wt.%-0.25 wt.%Sn. This improvement has been attributed to the formation of a Y-enriched oxide film on the alloy surface. Lin et al. [169] synthesized a bifunctional TiO2/Mg_2_TiO_4_ nano layer on the functionalized surface of a WE43 magnesium implant by using the dual titanium and oxygen plasma immersion ion implantation technique. A significant improvement in corrosion resistance of Mg substrates was reported together with the enhancement in the in vitro osteoblastic differentiation capability due to the controlled release of magnesium ions. Guo et al. [170] electrochemically synthesized a multifunctional polypyrrole/zinc oxide (Ppy/ZnO) composite coating on AZ31 Mg alloy by the cyclic voltammetry method. The multifunctional coating was reported to achieve a balance of good corrosion resistance, cytocompatibility, and excellent antibacterial property.

##### Ion Implantation and Stress Impartation Methods

V. K. Caralapatti and S. Narayanswamy [171] used High-Repetition Laser Shock Peening (HRLSP) to impart compressive residual stress on the surface of the Mg alloys to improve its degradation and biocompatibility. W. Jin et al. [172] carried out Nd ion implantation on WE43 using metal ion implanter with a Nd cathodic arc source. The retardation of the degradation rate and improvement in biocompatibility was attributed to a hydrophobic surface layer of mostly Nd_2_O_3_ and MgO. Y. Zhao et al. [173] used a dual Zirconium and Oxygen ion implantation method to create a hydrophobic surface film containing ZrO_2_ to achieve better cytocompatibility and corrosion resistance of the Mg-Ca and Mg-Sr alloys. It had the added advantage of having better antibacterial capability as well.

Most, if not all, the studies above focused on improving the surface characteristics of commercially available or common Mg alloys, and as such, there remains to be seen a concentrated effort in combining the development of high-performance biodegradable Mg alloys along with its surface modification tailored for specific applications/locations.

## 7. Effect of Implant Geometry on the Biodegradable Characteristics

Development of biodegradable Mg alloys in itself does not represent the practical application of it as an implant without discussing the physical dimensions of the alloy as an implant. This is because, despite the simplified models of a symmetric shape being used to test many of the degradation characteristics and mechanical performance in many of the studies, in practice, implants could come in a variety of complex shapes. Moreover, with regard to bone-grafting implants, the physical geometry and porosity of the implant are crucial factors in encouraging osteogenesis. Porosity is a result of enclosed/entrapped gaps within a sample. The more porous the alloy, the weaker the alloy is due to the stress concentration at these points. Additionally, the availability of gaps for the body’s contact fluid to seep into the implant offers an increased surface area for the degradation to take place, further weakening the implant. However, despite these inherent disadvantages, the complex implant geometry is a crucial aspect that is required for many bone-grafting and repair applications, and therefore the design of the implants needs to take into consideration the effect of its geometry on the final degradation characteristics and its subsequent deterioration of the mechanical properties. Mei Li et al. [174] reported the positive effects of hollow 3D implants for bone grafting [175]. Recently, additive manufacturing has gained focus in the fabrication of scaffolds exhibiting optimal porosity, fully interconnected structures, suitable compressive properties, and moderate corrosion behavior, thus meeting the basic requirements for tissue engineering scaffolds [176,177,178,179].

## 8. Prospects of Some of the Commercially Popular Mg Alloys

The commercially available Mg alloys have the chief advantage of being economically favorable due to the common availability of the alloys. These alloys were primarily made for industrial uses to take advantage of the high strength-to-weight ratio of the Mg alloys. Much research has been carried out to investigate the potential of these alloys as biodegradable implant materials. This includes their use as master alloys in casting and further processing of these alloys such as heat treatment, coating, etc. However, the chief disadvantage of some of these alloys is that since they have been developed for industrial use, they may contain one or more biotoxic elements, making them unpractical for use as a biodegradable material. Of these commercial alloys, those with aluminum are the most popular, and a number of studies have been carried out to determine its potential for use as a biodegradable Mg alloy.

An experiment conducted by Chen Ying Liang et al. [180] found that the corrosion resistance in increasing order is AZ91 < AZ31 < AM60 < ZK60 when tested on commercially available rolled plates of these Mg alloys. When tested in a 3.5% NaCl solution, the corrosion rate increased in terms of weight loss: Mg < AZ91 < AZ31, though after 3 h, the corrosion rate of AZ91 increased above that of AZ31, attributed to the start of galvanic corrosion [181]. The lower corrosion rate is attributed to the formation of Mg(OH)_2_ on its surface. In vitro tests conducted in SBF by J. Fu et al. [182] on as-cast Mg-Zn-Ca alloys concluded that micro-galvanic corrosion between the eutectic products of (Mg + Ca_2_Mg_6_Zn_3_) and the Mg matrix were the main form of corrosion. Hence, the biocorrosion rate increases with the increase in the volume fraction of the secondary phase. Additionally, they have also observed that large-grained samples corroded faster.

## 9. Conclusions

A wide range of Mg alloys are available with Al as the primary alloying element, which has a neurotoxic effect and causes Alzheimer’s disease. Reports indicate that the presence of silicon in these alloys helps to lower the accumulation of Al in mice and is perhaps conceivable to address the neurotoxic effect with an Mg-Al-Si alloy system, which needs more research both in vitro and in vivo.

Manufacturing processes such as ECAE/ECAP can be utilized in the future to achieve equiaxed grains in all directions, i.e., axially as well as radially. This could achieve better symmetry of tension vs. compression strength.

Various studies prove the degradation resistance enhancement of ‘as-made’ alloys with post processing operations. It is conceivable to address the issues related to the initial corrosion characteristics and its resultant weakening of the implant by adopting suitable heat treatment and surface modification regimes to achieve a very realistic clinical biodegradable Mg alloy.

The Mg alloys being developed and tested for application-specific implants need to be tested by clinical studies. Biodegradability is inherent in nature and, as such, the local environment of exposure would dictate the biological mechanisms in interaction with the implant alloy. Clinical trials of the biodegradable alloys are critically important as there is concern of the effect of the degradation product, which does not arise in in the case of non-biodegradable or inert implants.

With the various available biodegradable Mg alloys, each with its own set of characteristics, and the various available heat treatments, surface modifications, and coatings, the design of implants with functional gradations could provide a breakthrough in the clinical use of implants. For example, a high-strength, high-degradation core complemented by low degradation, yet relatively low strength can be used for osteogenesis applications requiring gap filling. This would ensure sufficient core strength of the implant while bone formation takes place, while, by the time the fast degradation of the core occurs, sufficient healing would have occurred to provide the final disintegration of the Mg implant.

## Figures and Tables

**Table 1:** Summary of toxicology of the common Mg alloying elements.

ASTM Code	Chemical Symbol	Whole Blood Level (Mean)			Blood Serum Levels (Mean)		General Daily Allowance for Adults (mg)		Toxicology and Pathophysiology
		French, μg/L [48] *	Elderly Swedish, (Mean ± SD) μM/L [49] ***	Benin, μg/L (Male) [50] **	French, μg/L [48] *	Elderly Swedish, (Mean ± SD) μM/L [49] ***	Male	Female	
	Mg	-	-	27858	-	-	260 [31]/400–420 [51]	220 [31]/310–320 [51]	Non-toxic except at high levels [31]. However, the upper limit is dependent on various factors such as gender/age/diet.
X	Ca	-	-	-	-	-	1000 [31,51]	1000–1300 [31,51]	An essential element of the body [31]. Makes up the human skeletal system.
C	Cu	1523	12.9 ± 1.91	874.925	1642	15.1 ± 2.92	0.9 [51]	0.9 [51]	It is an essential trace element of the body [34]. Above 3 mg/L of whole blood concentration of Cu leads to gastrointestinal symptoms of toxicity [34].
F	Fe	-	-	472457	-	-	8 [51]	18 [51]	Essential for normal metabolism of cells [33]. Have been reported to be toxic to cells under certain conditions [36].
M	Mn	33.8	0.144 ± 0.043	19.936	14.2	0.0284 ± 0.021	2.3 [51]	1.8 [51]	It is a trace element, Mn^2+^ is the predominant form in human body [52]. Leads to neurotoxic effects (manganism) only when exposed to inhalation [52]. Almost entirely excreted in the feces [52].
Z	Zn	6663	95.9 ± 12.7	4937.58	1529	11.2 ± 1.7	11 [51]	8 [51]	Zn is an essential [31] trace element [36]. The plasma zinc is regulated via homeostatic control [31].
J	Sr	9.6	-	31.792	23.8	-			Sr is not an essential element. Strontium ranelate is used for treatment of osteoporosis [53].
L	Li	0.268	-	0.474	5749	-	-	-	Non-Essential trace element without which the human body can lead a healthy life [46]. Long-term dosage and high dosage can be toxic. Excreted almost completely via kidneys with low tissue accumulation [46].
W	Y		-	-	-	-	-	-	Water-insoluble Y compounds are non-toxic but water-soluble compounds are mildly toxic [54]. Y and its compounds have been reported to have caused liver and lung damage in animals [54].
V	Gd	-	-	-	-	-	-	-	Highly toxic as a free ion [54]. It is used after chelation as MRI contrast agents. The strength of the chelation determines the toxicity [54].
A	Al	-	0.709 ± 0.539	3.726		0.424 ± 0.752	-	-	Aluminum is rated as Generally Regarded As Safe (GRAS) by US FDA [32]. However, concerns of its role in Alzheimer’s due to its accumulation in the brain exist, although the consensus for this is disputed [45].
N	Ni	18.8	0.144 ± 0.175	-	5.94	0.0446 ± 0.0527	-	-	Circumstantial evidence as an essential element [37]. Potentially leads to Cancer in forms administered other than orally [37].
B	Bi	4.72	-	<0.010	0.01	-	-	-	Found to be toxic in high doses [55].
Q	Ag	0.127	-	-	0.234	-	-	-	Ag is reported to be extremely toxic and is potentially fatal in the case of ingestion in the form of silver salts [56].
T	Sn	5.59	-	0.257	0.443	-	-	-	Not an essential element [57]. Found in cans and some vitamin supplements. Relatively non-toxic (barring respiratory forms), but in chronic doses, tends to accumulate in bones, kidney, and liver and may cause liver and kidney problems [57].
K	Zr	-	-	-	-	-	-	-	Zr dental implants have been found to be biocompatible with good osseointegration with good soft tissue response [58]. Majority is excreted via urine while absorption is dependent on the species of Zr [59].
S	S	-	-	-	-	-	-	-	Circumstantial evidence as an essential element has been reported [37]. Previously considered as biologically inert (breast implants), but exposure to severe and long-term doses could lead to inflammation of liver and spleen [42].
E	Nd	-	-	-	-	-	-	-	Low to moderate toxicity has been observed [54].
E	La	-	-	-	-	-	-	-	Animal tests involving injection of La in solution form has been reported to cause low blood pressure, hyperglycemia, hepatic alterations, and degeneration of the spleen [54].
E	Ce	-	-	<0.010	-	-	-	-	Experiments involving high dosage of Cerium injection in animals have led to fatal cardiovascular collapse [54].

* Reported from 106 Adults in a French hospital population (Male and Female). ** Arithmetic mean values reported for 70 healthy Males from Benin (Cotonou) with no occupational history of exposure to the tested elements. *** Reported from 1016 70-year-old Swedish population.

**Table 2:** In vitro cytotoxicity test results for various cell lines as found in literature.

Astm Code	Element	In Vitro Test Results	Reported Concentration	Cell Line(S)/Cell Description	Duration (Day)	Medium	Ref.
-	Mg	Cells were fully viable.	160 × 10^3^ ng/mL	U-2OS/(human osteosarcoma)			[63]
		Increased cell viability of cells. Viability was 171.1 ± 11.6% after day 5.	7 × 10^4^ cells/μL	hBMMSCs/(human bone marrow mesenchymal stem cells)	1, 3, 5	α-MEM	[4]
A	Al	Hemolysis and adhered platelets decreased for Mg-Al alloy as compared with Mg element.	20 ± 7 μM/L	Platelets	7	DMEM	[62]
		Al showed no decrease in cell viability		L929/NIH3T3/(fibroblasts) MC3T3-E1/(osteoblasts)			
		Al alloyed Mg showed no observed negative effects on cell viabilities.		ECV304/VSMC/blood vessel related cell			
X	Ca	Not toxic when Mg-1Ca was tested on cells. It also showed good viability.	-	L929	7	DMEM	[64]
C	Cu	Low Cu concentration, i.e., Cu wt.% of 0.03 and 0.19 stimulates growth of tested cells and promotes initial cell adhesion and spreading.	0.03–0.19 wt.%	HUVEC/MC3T3-E1	1	α-MEM and Endothelial cell medium, respectively	[65]
		High Cu concentration such as Cu wt.% above 0.57% is slightly toxic for cell proliferation.	>0.57%	HUVEC/(Human Umbilical Vein Endothelial cells) and MC3T3-E1	1–5		
F	Fe	Low iron concentrations are favorable for metabolism of cells.	<10 μg/mL	HUVEC/(Human Umbilical Vein Endothelial cells)	3	(Cell proliferation agent) WST-8	[66]
		No difference in metabolism of cells compared to zero Fe concentration.	50 μg/mL		1		
		High concentrations are cytotoxic to cells.	>50 μg/mL		1, 3		
M	Mn	Serious toxic effect to the tested cell lines.	1.8 μM/L	L929/(fibroblasts)	7	DMEM	[62]
				NIH3T3/(fibroblasts)			
				MC3T3-E1/(osteoblasts)			
				ECV304/blood vessel related cell			
				VSMC/blood vessel related cell			
S	Si	Increased cell viability of cells.	71 ± 27 μM/L	MC3T3-E1/(osteoblasts)			
		Toxic for the cells tested.		ECV304/VSMC/blood vessel related cell			
Z	Zn	Hemolysis and adhered platelets decreased for Mg-Zn as compared with Mg element.	2.6 ± 1 μM/L	Platelets			
		Mg-Zn showed no decrease in cell viability		L929/NIH3T3/(fibroblasts)			
				MC3T3-E1/(osteoblasts)			
				ECV304/blood vessel related cell			
		Zn alloyed Mg showed no observed negative effects on cell viabilities		VSMC/blood vessel related cell			
K	Zr	Serious toxic effect to the tested cell line	6.9 ± 1 μM/L	NIH3T3/L929/(fibroblasts)			
				ECV304/VSMC/blood vessel related cell			
				Platelets			
T	Sn	Hemolysis and adhered platelets decreased for Mg-Sn as compared with Mg element.	15.8 ± 7.8 μM/L	L929/NIH3T3/(fibroblasts)			
		Sn showed no decrease in cell viability.					
				MC3T3-E1/(osteoblasts)			
				VSMC/ECV304/blood vessel related cell			
		Showed negative effects on blood-vessel-related cell viabilities. Especially toxic from Mg-1Sn alloy extract.					
		Showed negative effects on blood-vessel-related cell viabilities.		ECV304 cells			
		Toxic at the tested concentration		MG63			
		Mg-1Sn, Mg-3Sn are harmless to tested cells.	1–3 wt.%	ATDC5	6		[67]
V	Gd	Good cell viability results when tested with ATDC5 cells [9]. Mg10Gd promoted cell maturation and hypertrophy in vitro indicating enhanced healing possibilities in vivo [9].	-	L929/(fibroblasts)	7	α-MEM + DMEM/F12-HAM	[68]
Q	Ag	Serious toxic effect to the tested cell lines.	0.9 ± 0.5 μM/L	NIH3T3/(fibroblasts)	7	DMEM	[62]
				MC3T3-E1/(osteoblasts)			
				ECV304/blood vessel related cell			
				ATDC5			
		Good cell viability when tested with ATDC5 cells.	-	L929/(fibroblasts)		α-MEM + DMEM/F12-HAM	[68]
	In	No significant viability changes.	5 ± 1.8 μM/L	NIH3T3/(fibroblasts)	7	DMEM	[62]
				MC3T3-E1/(osteoblasts)			
		Toxic for tested cells.		ECV304/VSMC/blood vessel related cell			
				MC3T3-E1/(Murine calvarial preosteoblasts)			
E	Nd and La	Cytotoxic for tested cells at high concentrations. Cytotoxicity decreased with lower concentrations.	-	MC3T3-E1/(Murine calvarial preosteoblasts)	5		[69]
	Ce	Severely cytotoxic for MC3T3-E1 cells even in low concentrations.	-				

DMEM: Dulbecco’s Modified Eagle Medium. α-MEM: Alpha- Minimum Essential Medium. F12-HAM: Nutrient Mixture (Sigma-Aldrich).

**Table 4:** A compilation of some of the favorable degradation rates of binary alloys of biodegradable Mg alloys along with their work history as found in literature.

Electrochemical Test											
Composition (wt.%)	Work History	Duration	Corrosion Potential		Corrosion Current Density		Corrosion Rate		Immersion Test, CR (Mass Loss)		Ref
			SBF	Hank’s	SBF	Hank’s	SBF	Hank’s	Hank’s	(DMEM + 10%FBS)	
		HRS	Ecorr (V)		Icorr (μA/cm^2^)		mm/yr				
Pure Mg	As-Cast	168	-	-	-	-	9.55 ± 1.19 ^P^	-	-	0.66 ± 0.36	[85]
		720	-	-	-	-	-		2.08 ± 0.2	-	[84]
		72	-	-	-	-	-		1.318681	-	[65]
		168	-	-	-	-	-		1.507064	-	
		500	1.886	1.533	86.06	15.98	1.94	0.36	-	-	[62]
	As-Rolled	500	1.796	1.544	37.24	9.58	0.84	0.22	-	-	
Mg–1Al	As-Cast	500	1.777	1.522	91.81	17.58	2.07	0.4	-	-	
			1.764	1.5	360.2	51.39	8.12	1.34	-	-	
	As-Rolled		1.685	1.391	136.8	172.9	3.09	3.9	-	-	
			1.708	1.514	53.95	26	1.22	0.59	-	-	
Mg–1In	As-Cast		1.905	1.561	103	19.48	2.32	0.44	-	-	
	As-Rolled		1.863	1.472	42.6	16	0.96	0.36	-	-	
Mg–1Mn	As-Cast		1.811	1.511	109.1	24.27	2.46	0.55	-	-	
	As-Rolled		1.825	1.486	20.15	5.71	0.45	0.13	-	-	
Mg–1Si	As-Cast		1.568	1.513	296	47.95	6.68	1.08	-	-	
	As-Rolled		1.634	1.452	28.36	21.17	0.64	0.48	-	-	
Mg–1Sn	As-Cast		1.893	1.621	108.8	16.3	2.45	0.37	-	-	
	As-Rolled		1.787	1.471	54.84	13.76	1.24	0.31	-	-	
Mg–1Y	As-Cast		1.703	1.49	140	27.67	3.16	0.62	-	-	
	As-Rolled		1.848	1.502	73.06	16.63	1.65	0.38	-	-	
Mg–1Zn	As-Cast		1.822	1.609	67.3	10.47	1.52	0.24	-	-	
	As-Rolled		1.805	1.549	40.78	7.55	0.92	0.17	-	-	
Mg–1Zr	As-Cast		1.886	1.55	97.69	21.73	2.2	0.49	-	-	
	As-Rolled		1.633	1.522	40.2	12.15	0.91	0.27		-	
Mg0.03Cu	As-Cast	72	-	-	-	-	-		10.54945	-	[65]
	As-Cast	168	-	-	-	-	-		9.230769	-	
Mg0.5Sr	As-cast	372 *	-	-	-	-	-	-	1.157076 ^H^	-	[110]
	Cast (Homogenized at 450 °C + Quenched)	372 *	-	-	-	-	-	-	0.777605 ^H^	-	
	Cast (Aged 150 °C, 360 h + Quenched)	372 *	-	-	-	-	-	-	0.827372 ^H^	-	
Mg–0.5Ca	As-Cast	84	−1.986 ^K^		186 ^K^		1.52 ^K^			-	[86]
Mg-Fe (Mg_30_Fe_70_)	Ball-milled, SPS (500 °C, 600 MPa, 10 min)	240	-	-	-	-	-	-	0.00292 ^d^	-	
Mg-0.69La	As-Cast	250	-	-	-	-	14.7 ± 0.92 **	-	-	-	[69]
Mg-1.27Ce			-	-	-	-	9.6 ± 0.78 **	-	-	-	
Mg-2.13Nd			-	-	-	-	4.1 ± 0.29 **	-	-	-	

* including the 12 h pre-corrosion exposure to corrosive media. ** as derived from H_2_ evolution rates from immersion test (included due to scarcity of other corrosion tests of binary alloys of the respective elements). ^H^—HBSS immersed. ^K^—Kokubo solution. ^P^—PBS: Dulbecco’s Phosphate Buffered Saline without Calcium and Magnesium salts. ^d^—Calculated as per ASTM-G1-72 for 1.6% weight loss during immersion period.

**Table 5:** A summary of the mechanical properties of some of the Mg binary alloys as reported in various literature.

Materials	YS, MPa			UTS, MPa			UCS, MPa			Elongation, %			
	As-Cast	As-Rolled	As-Extruded	As-Cast	As-Rolled	As-Extruded	As-Cast	As-Rolled	As-Extruded	As-Cast	As-Rolled	As-Extruded	Ref
Mg	20.83	113.2	-	86.69	169.6	-	-	-	-	13.06	12.26	-	[62]
Mg-1Al	42.34	168.8	-	159.94	230.1	-	-	-	-	16.58	6.09	-	
Mg-1Ag	23.86	126.9	-	116.26	196.6	-	-	-	-	13.34	6.687	-	
Mg-1In	35.62	133.5	-	145.82	191.6	-	-	-	-	14.96	9.473	-	
Mg-1Mn	28.9	116.5	-	82.99	172.1	-	-	-	-	7.536	3.741	-	
Mg-1Si	80.3	120.7	-	194.21	196.1	-	-	-	-	14.85	3.582	-	
Mg-1Sn	35.28	146	-	149.18	203.2	-	-	-	-	20.04	6.647	-	
Mg-1Y	25.54	146.8	-	74.59	199.9	-	-	-	-	9.992	9.154	-	
Mg-1Zn	25.54	160.5	-	133.39	239.7	-	-	-	-	18.25	7.124	-	
Mg-1Zr	67.2	131	-	172.03	182.9	-	-	-	-	27.02	17.27	-	
Mg-1Ca	40.26	123.7	136.2	71.54	166.8	240.13	-	-	-	1.911	3.196	10.81	
Mg-0.57Cu	-	-	-	104.14	-	-	167.48	-	-	-	-	-	[65]

## Data Availability

Not applicable.
